# 

**Published:** 1948

**Authors:** 


### Local Medical Notes

**The Carey Coombs Memorial Lecture** will be given on Thursday, January 27th, 1949, at 8.30 p.m., at the University, by Sir John Parkinson, M.D., F.R.C.P. The subject of the lecture will be " The Radiology of Rheumatic Heart Disease ".

### UNIVERSITY OF BRISTOL.

The following appointments have been conferred on former students and members of staff :

J. Benn, Honorary Physician, Bridgwater Hospital.

Geoffrey Hadfield, Sir William H. Collins Professor of Human and Comparative Pathology, Royal College of Surgeons.

R. B. Pickles, Dental Officer to the Ministry of Health.

P. J. Stoy, F.D.S.R.C.S., Professor of Dentistry, Queen's University, Belfast.

W. Hobson, Professor of Social and Industrial Medicine, University of Sheffield.

The following appointments have been made: F. Blakemore, *Professor of Veterinary Medicine.* G. L. Alexander, *Surgeon-in-Charge of the University Neurosurgical Unit.* D. G. Phillips, *Surgeon, University Neurosurgical Unit.*

### SCHOLARSHIPS AND PRIZES.

*Beaverbrook Fellowship.*—H. D. Moore.
*Royal Hospital Board's Gold Medal.*—P. W. Seargeant.
*Royal Hospital Board's Silver Medal.*—W. J. Gall.
*Martyn Memorial Scholarship.*—G. Walters.
*Open Entrance Scholarships.*—G. A. Bond, Winifred J. Hobbs.
*Edward Fawcett Memorial Prize.*—P. I. Draper.
*Thomas Francis Edgeworth and Francis Henry Edgeworth Prize.*—June K. Lloyd.
*Foyle Prize.*—June K. Lloyd.
*Mackie Pathology Prize.*—A. J. Lee.
*David Rayner Prize.*—Suzanne K. R. Clarke.
*Ophthalmology Prize.*—D. J. Brain.
*Dental Gold Medal.*—G. H. G. Jennings.
*De Trey Prize.*—D. Clifford.
*George Fawn Prize.*—L. K. Walden.
*Chadwick Medal.*—William Nicol.

### EXAMINATION RESULTS.

Members of the University have been awarded the following degrees and diplomas :

**University of Bristol.**

M.D.—J. N. P. Davies (with Distinction), Cecil M. Drillien, G. H. Wattley, D. N. Walder.

M.D.S.—J. W. E. Snawdon.

D.P.H.—D. M. M. Jones, E. W. Moore.

D.M.R.D.—W. R. Cole.

**University of Cambridge.**

B.A.—E. J. Watson-Williams.

**Royal College of Surgeons.**

F.R.C.S.—T. J. Butler, A. N. H. Peach, P. R. Slade.

**Royal College of Physicians.**

M.R.C.P.—P. Jacobs, G. H. Wattley.

**Royal College of Obstetrics and Gynaecology.**

M.R.C.O.G.—S. D. Loxton.

**Royal College of Physicians and Surgeons.**

D.A.—Violet Fry.

D.C.H.—M. P. Bourke, Sheila R. Tangye.

